# Draft Genome Sequence of *Trueperella bernardiae* LCDC 89-0504^T^, Isolated from a Human Blood Culture

**DOI:** 10.1128/genomeA.01634-15

**Published:** 2016-02-25

**Authors:** Anne-Marie Bernier, Kathryn Bernard

**Affiliations:** a Department of Biology, Université de Saint-Boniface, Winnipeg, Canada; b National Microbiology Laboratory CSCHAH Site, Public Health Agency of Canada, Winnipeg, Canada; c Department of Medical Microbiology, University of Manitoba, Winnipeg, Canada

## Abstract

We report here the draft genome sequence of *Trueperella bernardiae* LCDC 89-0504^T^, an organism linked to mild to severe infections in humans and animals. The genome size is 2,028,874 bp, with a G+C content of 65.44%. Annotation of the genome revealed 5 rRNA sequences, 48 tRNA genes, and 1,762 coding sequences.

## GENOME ANNOUNCEMENT

The catalase-negative and Gram-positive coccobacillus *Trueperella bernardiae* is an infrequent and opportunistic pathogen in both humans and animals (1). Infections by *T. bernardiae* in humans range from mild to severe, including brain abscess (2) and necrotizing fasciitis (3). Nearly 30 years ago, bacteria recovered from blood cultures, bone, wounds, abscesses, and skin infections and found to be similar after biochemical testing were first described using the provisional name CDC fermentative Coryneform group 2 (CDC group 2) (4). CDC group 2 was formally assigned to *Actinomyces bernardiae* sp. nov., based on 16S rRNA gene sequencing and other features for strains recovered from infections in Canada, the United States, and Switzerland (5). In 1997, *A. bernardiae*, along with *A. pyogenes* and *A. phocae* sp. nov., were assigned to the genus *Arcanobacterium*, based on reanalysis of the 16S rRNA gene sequences and subsequent topography after comparison with species in the genus *Actinomyces* (6). This species was later reassigned to *Trueperella* gen. nov., along with *A. abortisuis, A. bialowiezensis, A. bonasi* and *A. pyogenes*, based on further reassessment of phylogenetic positioning and chemotaxonomic characteristics (7). Here, we describe features derived from whole-genome sequence analysis of *T. bernardiae* LCDC 89-0504^T^. Health Canada’s Laboratory Centre for Disease Control (LCDC), located in Ottawa, Ontario, is now called the National Microbiology Laboratory and is located in Winnipeg, Manitoba, Canada. LCDC 89-0504^T^ has also been deposited as ATCC 51727^T^ (= CCUG 33419^T^ = CIP 104252^T^ = DSM 9152^T^ and LMG 18721^T^).

DNA from *T. bernardiae* was purified using the TruSeq DNA HT sample preparation kit, as per the manufacturer’s protocol. The whole-genome shotgun library was sequenced in a paired-end run using the MiSeq sequencer (Illumina 1.9, 2 × 300 cycles), according to the manufacturer’s protocols. Sequencing generated 1,501,256 reads and 451,878,056 detected bases. Overlapping paired-end reads were merged using Fast Length Adjustment of SHort reads (FLASH) (8) to produce longer single reads that were subsequently assembled with paired-end reads using the SPAdes genome assembler (St. Petersburg genome assembler, version 3.5.0) (9), using k-mer values of 21 to 127. This generated 57 contigs, which were subsequently filtered to remove repeated sequences, leaving 18 contigs (>1,000 bp) with an *N*_50_ of 310,371 bp. The average contig size was 112,715 bp, with an average coverage of 110-fold.

The genome size of *T. bernardiae* is 2,028,874 bp, with a G+C content of 65.44% (63 to 66% by the thermal denaturation method [7]). Gene prediction and annotation were performed with Prokka (10). There are 1,816 genes with 1,762 coding sequences, 5 rRNA genes, 48 tRNA genes, and 1 transfer-messenger RNA (tmRNA). The genome was found to harbor one clustered regularly interspaced short palindromic repeat (CRISPR) sequence.

### Nucleotide sequence accession numbers.

The whole-genome shotgun project for *T. bernardiae* LCDC 89-0504^T^ has been deposited at DDBJ/EMBL/GenBank under the accession no. LNIZ00000000. The version described in this paper is LNIZ01000000.
